# Circular RNAs: Characteristics, Function and Clinical Significance in Hepatocellular Carcinoma

**DOI:** 10.3390/cancers10080258

**Published:** 2018-08-02

**Authors:** Man Wang, Fei Yu, Peifeng Li

**Affiliations:** Institute for Translational Medicine, Medical College of Qingdao University, Dengzhou Road 38, Qingdao 266021, China; wangman@qdu.edu.cn (M.W.); yufei917@126.com (F.Y.)

**Keywords:** HCC, circRNA, miRNA sponge, diagnostic biomarker, therapeutic target

## Abstract

Hepatocellular carcinoma (HCC) is one of the leading causes of cancer-related deaths worldwide. HCC patients are commonly diagnosed at an advanced stage, for which highly effective therapies are limited. Moreover, the five-year survival rate of HCC patients remains poor due to high frequency of tumor metastasis and recurrence. These challenges give rise to the emergent need to discover promising biomarkers for HCC diagnosis and identify novel targets for HCC therapy. Circular RNAs (circRNAs), a class of long-overlook non-coding RNA, have been revealed as multi-functional RNAs in recent years. Growing evidence indicates that circRNA expression alterations have a broad impact in biological characteristics of HCC. Most of these circRNAs regulate HCC progression by acting as miRNA sponges, suggesting that circRNAs may function as promising diagnostic biomarkers and ideal therapeutic targets for HCC. In this review, we summarize the current progress in studying the functional role of circRNAs in HCC pathogenesis and present their potential values as diagnostic biomarkers and therapeutic targets. In-depth investigations on the function and mechanism of circRNAs in HCC will enrich our knowledge of HCC pathogenesis and contribute to the development of effective diagnostic biomarkers and therapeutic targets for HCC.

## 1. Introduction

Hepatocellular carcinoma (HCC), the most common malignancy of the liver, ranks the third leading cause of cancer-related death worldwide and has a significantly low survival rate [1,2]. HCC causes at least 700,000 deaths every year around the world [3]. The high metastatic capability and recurrence rate of HCC result in low survival rate of HCC patients. Therefore, HCC has become a severe public health problem worldwide. It has been found that hepatitis B virus (HBV) and hepatitis C virus (HCV) infections are the major risk factors of HCC [4]. So far, common therapeutic strategies for HCC include chemotherapy, liver transplantation, surgical resection, anti-tumor drug therapy, and radiation [5,6]. HCC patients may achieve marked advantage in life expectancy if they are diagnosed at an early stage. Nevertheless, most HCC patients are usually diagnosed at an advanced stage with metastasis before any curative treatment can be applied. Thus, the early diagnosis of HCC is a main issue that remains to be solved. Moreover, there is no effective strategy available for HCC prevention. Therefore, it is urgently needed to explore the molecular mechanism underlying HCC tumorigenesis and to identify valuable diagnostic biomarkers and therapeutic targets for HCC.

Non-coding RNAs (ncRNAs) are functional RNAs that are generally not translated into proteins [7]. ncRNAs constitute a wide diversity of RNA molecules with different structural and functional characteristics. According to the transcript length, ncRNAs are mainly classified into two classes: short ncRNAs (<200 nucleotides) and long ncRNAs (lncRNAs, >200 nucleotides) [8]. Several types of short ncRNAs have attracted many investigations such as microRNAs (miRNAs), piwi-interacting RNAs (piRNAs), and small interfering RNAs (siRNAs) [9,10]. Increasing evidence indicates that these subtypes of short ncRNAs and lncRNAs play crucial regulatory roles in a variety of biological and cellular processes. Their best characterized function is regulation of gene expression. For instance, miRNAs can specifically bind to target mRNAs to cause mRNA degradation or inhibit protein translation [11,12], while lncRNAs are able to interact with DNAs, RNAs, or proteins and control gene expression at transcriptional and post-transcriptional levels [13,14,15]. More importantly, miRNAs and lncRNAs are commonly dysregulated in HCC, highlighting the significance of ncRNAs in the tumorigenesis and progression of HCC [16,17]. 

Notably, circular RNAs (circRNAs) are a newly discovered type of ncRNAs and ubiquitously exist in many species [18,19]. Unlike the canonical linear RNAs, circRNAs form a covalently closed continuous loop structure with neither 5′ caps nor 3′ polyadenylated tails, which makes them more stable than linear RNAs [20]. They are generated through non-sequential back-splicing of pre-mRNAs, in which a downstream splice donor is linked to an upstream splice acceptor [21]. CircRNAs are initially regarded as byproducts or spliced intermediates of errant RNA splicing events. With the advancement of high-throughput sequencing and bioinformatics approaches, a number of circRNAs have been identified in viruses, fungi, plants and animals [22,23,24,25,26]. Further studies show that circRNAs can function as molecular sponges for miRNAs and RNA-binding proteins (RBPs) [27,28,29,30]. CircRNAs are also vital regulators in gene transcription and expression [31]. Furthermore, circRNAs are highly conserved across multiple species and exhibit tissue-specific and development stage-dependent expression patterns [32,33]. These features imply that circRNAs possess significant functions in biological and pathological processes. Currently, there is a growing body of literature showing that circRNAs are differentially expressed in HCC and serve a central role in the carcinogenesis and progression of HCC [34,35,36], demonstrating that circRNAs may represent promising diagnostic markers and therapeutic targets for HCC. In this review, we briefly summarize the characteristics and functions of circRNAs with emphasis on their functional role in biological processes associated with HCC tumorigenesis. We also discuss the potential values of circRNAs in HCC diagnosis and treatment. A better understanding of the function and mechanism of circRNAs in HCC tumorigenesis may contribute to the development of novel detection methods and effective therapeutic measures for HCC diagnosis and treatment.

## 2. Biogenesis of CircRNAs

**Figure 1:**
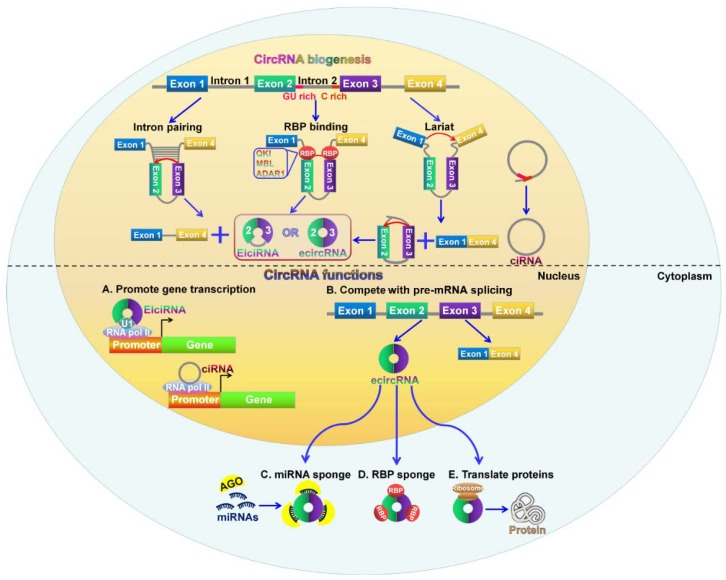
Biogenesis and functions of circular RNAs (circRNAs). The biogenesis of circRNAs is mainly regulated by three different mechanisms: intron pairing-driven circularization, RNA-binding proteins (RBPs)-mediated circularization, and lariat-driven circularization. CircRNAs can sequester and absorb miRNAs to regulate the function of miRNAs. CircRNAs also interact with proteins and thus regulate their cellular localization and activity. CircRNAs promote the transcription of their parental genes by interacting with RNA polymerase II (Pol II) or U1 small nuclear ribonucleoprotein (snRNP). CircRNAs play an important role in gene regulation by competing with canonical splicing of pre-mRNAs. Intriguingly, some circRNAs are capable of encoding proteins.

CircRNAs are produced through back-splicing events from exons of protein-coding genes, introns, intergenic regions, antisense, or untranslated regions [26,37,38]. The backsplicing process involves exon circularization between a downstream splice donor and an upstream splice acceptor [39]. Based on their origin, circRNAs are mainly classified into three categories: exonic circRNAs (ecircRNAs), exon-intron circRNAs (EIciRNAs), and circular intronic RNAs (ciRNAs) [40]. The three types of circRNAs are produced from different circularizing mechanisms (Figure 1). EcircRNAs are originated from exons and account for the majority of identified circRNAs [41]. Two models of ecircRNA formation have been proposed, including lariat-driven circularization and intron pairing-driven circularization [32]. In the lariat-driven circularization model, the non-adjacent exons in the pre-mRNA are in close proximity, forming a lariat intermediate consisting of several exons and introns. The introns are removed, followed by the connection between the downstream 5′ splice site (splice donor) of exons and the upstream 3′ splice site (splice acceptor). This connection results in the formation of ecircRNAs. In some cases, the introns present in the lariat are not spliced out completely but are retained within the encircled exons, resulting in the generation of EIciRNAs. In the intron pairing-driven circularizing model, a circular structure is formed via base-paring between reverse complementary sequences (e.g., Alu repeats) across exon-flanking introns. Intron paring is followed by back-splicing of pre-mRNAs and exon circularization.

Additionally, RNA-binding proteins (RBPs) act as trans-factors involved in circRNA biogenesis. For instance, the alternative splicing factor, Quaking (QKI), can bridge the 5′ splice site closer to the upstream 3′ splice site by binding to flanking introns, thus promoting ecircRNA formation [18]. Similarly, muscleblind (MBL) can bridge two flanking introns closer together by binding to its own pre-mRNA, promoting circRNA generation from its own RNA [21]. The RNA-editing enzyme adenosine deaminase acting on RNA 1 (ADAR1) exerts inhibitory functions in circRNA formation [42]. Mechanistically, the function of ADAR1 in circRNA formation is correlated with its Adenosine-to-Inosine (A-to-I) editing capability. ADAR1 can specifically targets dsRNA pairing structures and catalyze the conversion of A to I, destabilizing RNA paring and inhibiting backsplicing for circRNA production. 

Most introns are spliced out from pre-mRNAs and form a lasso structure that is degraded following off-branch [43]. ciRNAs are generated from intron lariats that escape the usual intron debranching and degradation processes [38]. It is known that the formation of ciRNAs depends on conserved motifs at both ends of the intron, such as a seven nucleotide GU-rich motif located at the 5′ splicing site and an 11 nucleotide C-rich motif at the 3′-branch site. These motifs can prevent intron debranching and cause the intron to form a circular structure. The resulting RNA circle is covalently linked through a 2′,5′-phosphodiester bond at the junction site. The 3′ tail stretching from the 3′ end of the intron to the branch point is removed to produce stable ciRNAs.

## 3. Biological Function of CircRNAs

CircRNAs have become a new star in the field of ncRNA research. The biological function of circRNAs has been extensively investigated. Their function can be grouped into five parts: serving as miRNA sponges to suppress their function, working as transcriptional and translational regulators, influencing alternative splicing of pre-mRNAs, interacting with RBPs to regulate gene expression, and having the potential to encode proteins (Figure 1).

### 3.1. CircRNAs can Act as miRNA Sponges

miRNAs function as gene expression regulators by directly binding mRNAs [44]. The interaction between miRNAs and mRNAs causes the suppression of mRNA translation or degradation of target mRNAs [45]. CircRNAs are found to contain miRNA response elements (MREs), demonstrating that circRNAs can competitively bind miRNAs [46]. Increasing evidence has shown that circRNAs can function as competitive endogenous RNAs (ceRNAs) or miRNA sponges to inhibit miRNA function [27]. The human cerebellar degeneration-related protein 1 transcript (CDR1as or ciRS-7) harbored more than 70 conserved miR-7 binding sites [26]. CDR1as bound miR-7 to favor the specific miR-7/AGO2 interaction, thus affecting the availability of miR-7 to attach to its target mRNAs. The circular form of sex-determining region Y (Sry) was generated through intron pairing-driven circularization process [47,48]. Sry circRNA contained 16 binding sites for miR-138 [27]. CircHIPK3 exhibited a promotive function in cancer cell growth by binding to multiple miRNAs including the tumor suppressor miR-124 [49,50]. Previous studies indicated that circ-ITCH could promote the expression of its parental cancer-suppressive gene *ITCH* by sponging miR-7, miR-17, and miR-214 [51,52]. Moreover, circ-ITCH-mediated ITCH upregulation suppressed Wnt/β-catenin signaling pathway. These results demonstrated that circ-ITCH had an inhibitory effect on cancer progression by increasing the expression of its parental gene. Additionally, circFGFR4 has been reported to promote myoblast differentiation [53]. CircFGFR4 could sponge miR-107, and its upregulation enhanced the expression of the miR-107 target Wnt3a. Therefore, circFGFR4 promoted cell differentiation via sponging miR-107 to diminish its inhibition of Wnt3a.

An increasing number of studies demonstrate that the miRNA sponge function of circRNAs is conserved among various species. Thus, the action of circRNAs as miRNA sponges is a common phenomenon. In some cases, the interaction between circRNAs and miRNAs may not always cause the inhibition of miRNAs. For instance, CDR1as was reported to bind miR-671 and miR-7 [54,55,56]. The interaction between CDR1as and miR-671 could induce the linearization and AGO2-mediated cleavage of CDR1as, resulting in the release of bound miR-7. Thus, circRNAs with miRNA-binding sites may serve as reservoirs or transporter of miRNAs. More detailed studies are required to elucidate the effect of circRNA/miRNA interaction on the expression and cellular localization of miRNAs.

### 3.2. CircRNAs Function as Transcriptional and Translational Regulators

The function of circRNAs in regulating gene expression has been shown in several studies. EIciRNAs retain the intronic sequences from their parental gene and thus are able to interact with the transcription machinery. For example, circEIF3J and circPAIP2 could interact with U1 small nuclear ribonucleoprotein (snRNP) and RNA Pol II in the promoter region of the host gene, thus enhancing the expression of their parental genes, eukaryotic translation initiation factor 3J (EIF3J) and poly(A)-binding protein-interacting protein 2 (PAIP2) [57]. CiRNAs mainly accumulate in the nucleus and are able to modulate gene transcription in *cis*. *Ci-ANKRD52*, *ci-MCM5* and *ci-SIRT7* were able to interact with the elongating Pol II complex [38]. Depleting these ciRNAs led to decreased transcriptional levels of their parental genes, *ankyrin repeat domain 52* (*ANKRD52*), *minichromosome maintenance complex component 5* (*MCM5*), and *sirtuin 7* (*SIRT7*). These results demonstrated that ciRNAs could promote transcription of their parental genes by regulating elongating Pol II activity.

In addition, circRNAs are found to work as modulators in the translational process of mRNA. The mouse *formin* (*Fmn*) gene could generate circRNAs via backsplicing [58]. The circRNAs harbored the translation start site, making the truncated linear mRNA incapable of being translated into Fmn protein. Depleting specific exons caused a failure to produce these circular transcripts but resulted in aberrant expression of the Fmn protein. This study indicated that circRNAs could function as mRNA traps by capturing the translation start site to modulate mRNA translation and protein expression. Taken together, circRNAs play an important role in regulating gene expression. However, the mechanisms underlying circRNA-mediated gene regulation are required to be further investigated.

### 3.3. CircRNAs Compete with Linear Splicing of Pre-mRNAs

CircRNAs are mainly generated from exons of protein-coding genes [59]. During pre-mRNA splicing, the formation of circRNAs via exon circularization can compete with canonical splicing of pre-mRNAs into linear RNAs since they work on the same splice sites [21]. A circularized exon means that it will not be implicated in the processed mRNA, suggesting that circRNAs are generally produced in expense of canonical splicing to generate mRNAs [60]. Moreover, the canonical spliceosomal machinery also functions in circRNA biogenesis [61]. There are also competitions between circRNA biogenesis and linear splicing of the pre-mRNA owing to the overlapping dependence on the spliceosomal machinery [39,62]. As a result, circRNA biosynthesis results in decreased production of their linear transcripts, demonstrating the negative correlation between circRNAs and their linear isoforms [63]. Therefore, circRNA formation affects alternative splicing of the pre-mRNA, contributing to altered gene expression. However, the molecular mechanisms involving the competition between circRNA generation and linear splicing of the pre-mRNA need more systematic survey.

### 3.4. CircRNAs can Interact with RBPs to Regulate Gene Expression

CircRNAs can bind to RBPs and function as RBP sponges. CircRNAs might sequester and transport RBPs to particular subcellular locations [64]. For example, circ-Amotl1 was capable of binding to c-myc, STAT3, PDK1, and AKT1 to facilitate their translocation into the nucleus, thus regulating the expression of their target genes [65,66,67]. A circRNA (circMbl) was found to be derived from the splicing factor *muscleblind* (*MBL*) locus, and its flanking introns harbored binding sites for the MBL protein [21]. CircMbl biogenesis was relied on the MBL binding sites within the introns flanking circMbl. The introns flanking circMbl had multiple MBL binding sites, which were vital for the circularization of bracketed exons dependent on MBL levels. When MBL protein was in excess, it might reduce the production of its own mRNA by promoting circMbl formation. The enriched circMbl could sponge out the excess MBL protein to decrease its protein level. On the other hand, circRNAs can modulate translation of its cognate mRNA by binding to translational activators. The translational activator HuR interacted with circPABPN1 that originated from the *nuclear poly(A)-binding protein 1* (*PABPN1*) pre-mRNA [29]. The extensive binding of circPABPN1 to HuR prevented HuR interacting with *PABPN1* mRNA, thereby inhibiting the translation of *PABPN1* mRNA.

### 3.5. CircRNAs Possess Translational Ability

The majority of circRNAs are composed of exons; thus, they may be translated into proteins. Recent studies confirm that circRNAs can encode for proteins. When an internal ribosome entry site (IRES) was introduced to the circRNA sequence, circRNAs could be translated in vivo and in vitro, indicating that circRNAs might have the potential to encode proteins [68,69]. Circ-ZNF609 contained a 753 nucleotides open reading frame (ORF) spanning from the start codon to an in-frame stop codon [70]. Circ-ZNF609 could encode two protein isoforms through a splicing-dependent and cap-independent manner, providing the first indication that endogenous circRNAs have translational capability. Circ-FBXW7 was found to be upregulated in human brains and encoded a 21 kDa protein termed FBXW7-185aa, which inhibited proliferation and cell cycle acceleration in cancer cells [71]. Another study indicated that the incorporation of N6-methyladenosine (m6A), the most abundant base modification of RNA, was sufficient to promote the initiation of protein translation from circRNAs in human cells in the presence of translational initiation factors eIF4G2 and YTHDF3 [72].

The biological function of circRNAs has been unveiled in recent years. As mentioned above, circRNAs can serve as molecular sponges to sequester miRNAs or proteins, and may influence their cellular abundance and localization. CircRNAs can regulate gene expression by sponging miRNAs/RBPs or competing with pre-mRNA splicing. Some circRNAs can even encode proteins. These studies suggest that circRNAs play an important role in various biological processes. However, the function of circRNAs remains largely unknown, and only a handful of studies elaborately disclose the biological functions of circRNAs. More efforts should be put into exploring the function of circRNAs.

## 4. The Functional Roles of CircRNAs in HCC

Increasing evidence indicates that a great number of circRNAs are aberrantly expressed in HCC tissues, suggesting that these circRNAs may perform a function in the carcinogenesis and development of HCC. The expression and function of deregulated circRNAs in HCC are listed in Table 1.

### 4.1. CircRNAs Act as Oncogenes in HCC

CircRNAs are expressed in the tissue-specific manner, implying that circRNAs may be involved in the development of various diseases [94,95,96]. Among them, the oncogenic circRNA, CDR1as, was shown to be deregulated in a variety of cancers, including HCC [97]. A previous study indicated that the high expression of CDR1as in HCC tissues was strikingly associated with hepatic microvascular invasion (MVI) and partly correlated with the deterioration of HCC [73]. This study indicated that CDR1as served as a risk factor for MVI in HCC. Another report showed that CDR1as was highly expressed in HCC tissues compared with adjacent non-tumorous tissues [74]. Knockdown of CDR1as inhibited the proliferation and invasion of HCC cells. CDR1as was shown to serve as a sponge of miR-7. Overexpression of miR-7 restrained the proliferation and invasion of HCC cells and also reduced the expression of their target genes, cyclin E1 (*CCNE1*) and phosphoinositide 3-kinase catalytic subunit δ (*PIK3CD*). Mechanistically, CDR1as promoted the proliferation and invasion of HCC cells by sponging miR-7 and then interfering with the PIK3CD/phospho-p70 S6 kinase (p70S6K)/the mammalian target of rapamycin (mTOR) signaling pathway. These findings demonstrated that CDR1as functioned to regulate HCC progression. CDR1as-regulated proteins were also identified in HCC cells by applying the quantitative proteomics-based strategy [75]. The proteomic analysis combined with functional verification indicated that CDR1as overexpression could promote the proliferation and cell cycle progression of HCC cells partly via modulation of epidermal growth factor receptor (EGFR) signaling by controlling miR-7 expression. 

Tumor cells initially undergo epithelial-mesenchymal transition (EMT), as characterized by loss of E-cadherin and gain of vimentin, to become metastatic and invasive [98,99]. The EMT-inducing transcription factor, Twist1, was found to upregulate the expression of Cul2 circRNA (circ-10720) [76]. Circ-10720 was positively associated with tumor malignance and poor prognosis in HCC. Further study indicated that circ-10720 promoted cell proliferation, migration, and invasion in HCC. In terms of mechanism, Twist1 could increase the expression of vimentin by upregulating circ-10720, which sequestered a series of miRNAs directly targeting vimentin. Therefore, the Twist/circ-10720 pathway exerted a promotive effect on EMT progression in HCC. Conversely, circ-10720 silencing abolished the tumor-promoting activity of Twist1 in vitro and in vivo. These findings demonstrated that circ-10720 played an oncogenic function in HCC progression and might serve as a potential therapeutic target for HCC treatment. This study also provided novel insights into circRNA-based therapeutic strategies for HCC intervention. 

Aquaporin 3 (AQP3) plays a vital role in carcinogenesis and cancer progression [100]. AQP3 was dramatically upregulated in HCC tissues, and high expression of AQP3 was correlated with tumor progression, metastasis, and prognosis in HCC patients [101,102]. miR-124-3p was significantly downregulated in HCC and performed an inhibitory function in the proliferation and migration of HCC cells by targeting AQP3 [36]. Further study indicated that circHIPK3 acted as a miR-124-3p sponge and thus regulated AQP3 expression. CircHIPK3 could promote the proliferation and migration of HCC cells via the miR-124-3p/AQP3 axis. In vivo study confirmed that knockdown of circHIPK3 suppressed HCC growth. The role of another circRNA hsa_circ_0000673 in HCC progression has also been unraveled [77]. It has been found that hsa_circ_0000673 was highly expressed in HCC tissues. Hsa_circ_0000673 knockdown significantly inhibited the proliferation and invasion of HCC cells and suppressed tumor growth in vivo. Mechanistically, hsa_circ_0000673 acted as a sponge for miR-767-3p and thus enhanced the expression of its downstream effector SET. SET was reported to act as an oncogene in tumorigenesis, and aberrant expression of SET was associated with cancer progression [103,104]. Remarkably, SET was found to be upregulated in HCC and strongly associated with poor clinical outcomes [105]. Taken together, hsa_circ_0000673 promoted HCC malignance by modulating miR-767-3p/SET pathway.

Recently, hsa_circ_0067934 was reported to enhance the proliferation, migration, and invasion of HCC cells [78]. In terms of mechanism, hsa_circ_0067934 could inhibit the activity of miR-1324 and thus activated frizzled class receptor 5 (FZD5)/Wnt/β-catenin signaling pathway. In summary, hsa_circ_0067934 played a promotive role in HCC development and was a promising therapeutic target for HCC treatment. Guan et al. [79] also screened the circRNA expression profiles in HCC tissues and paired normal liver tissues. They identified a total of 1245 differentially expressed circRNAs in HCC tissues, including 756 upregulated circRNAs and 489 downregulated circRNAs. Among these circRNAs, hsa_circ_0016788 was identified as an upregulated circRNA in HCC tissues. Loss-of-functional studies indicated that hsa_circ_0016788 knockdown suppressed the proliferation and invasion of HCC cells and promoted cell apoptosis. In vivo study showed that hsa_circ_0016788 silencing inhibited HCC tumor growth. Moreover, hsa_circ_0016788 could sponge miR-486 that negatively regulated the expression of cyclin-dependent kinase 4 (CDK4). Receiver operating characteristics (ROC) analysis further demonstrated that hsa_circ_0016788 possessed high diagnostic value in HCC. Collectively, the hsa_circ_0016788/miR-486/CDK4 axis performed a modulatory role in HCC tumorigenesis, providing a novel therapeutic target for HCC.

CircRBM23 was found to be abundantly expressed in HCC tissues [80]. CircRBM23 upregulation was able to increase cell viability and elevate the migratory capability of HCC cells. Oppositely, circRBM23 silencing was shown to inhibit the proliferation and migration of HCC cells. Specifically, downregulation of circRBM23 enhanced the expression of miR-138 and decreased the levels of its target genes, vimentin and cyclin D3 (CCND3). Thus, upregulated circRBM23 functioned as an oncogene in HCC with downregulation of tumor suppressor miR-138. Huang et al. [81] analyzed a large-scale circRNA expression profile in HCC tissues and matched pericancerous tissues from four patients. They identified 226 differentially expressed circRNAs in HCC tissues, of which 189 were significantly upregulated and 37 were downregulated. Upregulation of hsa_circ_100338 was tightly associated with a low cumulative survival rate and metastatic progression in HCC patients. More importantly, hsa_circ_100338 served as a molecular sponge for miR-141-3p in HCC. Hsa_circ_100338 overexpression was shown to positively regulate migratory and invasive potential in HCC cells. miR-141-3p could antagonize the regulatory function of hsa_circ_100338, thereby suppressing HCC metastasis. These findings suggested that hsa_circ_100338 was a potential biomarker and therapeutic target for HCC diagnosis and treatment. On the other hand, miRNAs can regulate HCC progression by regulating the expression of circRNAs. The expression of miR-200b was low in HCC, while Ras homologue A (RhoA) and circ_000839 were expressed at a high level in HCC [89]. Correlation analysis showed that miR-200b was negatively correlated with RhoA and circ_000839, while RhoA was positively correlated with circ_000839. Functional study indicated that miR-200b inhibited the migration and invasion of HCC cells by decreasing the expression of RhoA and circRNA_000839.

### 4.2. CircRNAs Act as Tumor Suppressors in HCC

The expression of circRNA SMAD2 (circSMAD2) was lower in HCC tissues compared with adjacent normal tissues [82]. CircSMAD2 was strikingly associated with the differentiation degree of HCC tissues. Overexpression of circSMAD2 could inhibit the migration, invasion and EMT of HCC cells. miR-629 was verified to be the target of circSMAD2. Moreover, miR-629 could reverse the impact of circSMAD2 in HCC progression. Another circRNA, circC3P1, was also shown to be downregulated in HCC [83]. CircC3P1 overexpression significantly inhibited the proliferation, migration and invasion of HCC cells. Moreover, circC3P1 also suppressed HCC growth and metastasis in vivo. Notably, circC3P1 enhanced the expression of phosphoenolpyruvate carboxykinase 1 (PCK1) by sponging miR-4641 in HCC cells. Inhibition of PCK1 expression markedly attenuated the effect of circC3P1 on HCC cell proliferation, migration and invasion. Collectively, circC3P1 functioned as a tumor suppressor via promoting PCK1 expression by targeting miR-4641 in HCC. Hsa_circ_0005986 also functioned as a tumor suppressor in HCC carcinogenesis [35]. It has been found that the expression of hsa_circ_0005986 was reduced in HCC tissues. Knockdown of hsa_circ_0005986 led to the release of miR-129-5p and thus lowered the expression level of its target gene, *Notch1*. More importantly, hsa_circ_0005986 downregulation promoted the proliferation of HCC cells by driving cell cycle transition. Additionally, the low expression level of hsa_circ_0005986 was correlated with the clinicopathological characteristics of HCC patients including tumor size, MVI, and Barcelona Clinic Liver Cancer (BCLC) stage. Consequently, hsa_circ_0005986 may not only perform an inhibitory function in HCC tumorigenesis but may be a promising biomarker for HCC diagnosis as well.

Yu et al. [84] compared the expression of circRNAs between paired HCC and adjacent non-tumorous tissues by using RNA-sequencing technology. They characterized one circRNA derived from exons 15 and 16 of the *SMARCA5* gene and named it cSMARCA5 (hsa_circ_0001445). They explored the functions of cSMARCA5 in HCC progression. The result indicated that the expression of cSMARCA5 was reduced in HCC tissues. The downregulation of cSMARCA5 in HCC was associated with aggressive clinicopathological characteristics and might work as a risk factor for overall survival and recurrence-free survival in HCC patients after surgical resection. cSMARCA5 overexpression was shown to inhibit the proliferation and migration of HCC cells. In terms of mechanism, cSMARCA5 enhanced the expression of tissue inhibitor of metalloproteinase 3 (TIMP3), a well-known tumor suppressor, by sponging miR-17-3p and miR-181b-5p. These results demonstrated the implication of cSMARCA5 in the growth and metastasis of HCC and provided a new perspective on the role of circRNAs in HCC development. Another study also reported the expression of hsa_circ_0001445 in HCC and matched pericancerous tissues [85]. The results indicated that the expression of hsa_circ_0001445 was significantly reduced in HCC tissues and correlated with the number of tumor foci. Gain-of-functional studies indicated that upregulation of hsa_circ_0001445 could promote cell apoptosis and inhibited the proliferation, migration and invasion of HCC cells in vitro, indicating that hsa_circ_0001445 exerted a regulatory function in HCC development. 

Recently, the expression profile of circRNAs in HCC tissues has been reported [86]. Among the differentially expressed circRNAs, circMTO1 was identified as a dramatically downregulated circRNA in HCC tissues as compared to that in matched adjacent liver tissues. The expression of circMTO1 was positively correlated with the survival of HCC patients. CircMTO1 overexpression was shown to suppress the proliferation and invasion of HCC cells in vitro. Further study indicated that circMTO1 functioned as a molecular sponge for miR-9 to abolish miR-9-mediated silencing of the tumor suppressor cyclin-dependent kinase inhibitor 1 (p21). Therefore, circMTO1 might suppress HCC progression partially by abrogating miR-9 oncogenic activity. Additionally, circMTO1 knockdown promoted HCC growth in vivo. These results suggested that circMTO1 might be a promising therapeutic target for HCC treatment and a novel biomarker for HCC prognosis.

ADAR1 has been reported to be an important regulator of circRNA biogenesis [42]. Abnormal A-to-I editing by ADAR1 promoted carcinogenesis in a variety of tissues [106]. Specifically, the aberrant expression of ADAR1 was shown to promote HCC progression [107,108]. Previously, the influence of androgen receptor (AR) on circRNA expression in HCC was studied [87]. AR transcriptionally enhanced ADAR1 expression by binding to its promoter. As a result, AR was found to suppress circRNA expression in HCC through ADAR1 that could directly block circRNA biosynthesis. Furthermore, circARSP91, derived from *polyadenylate-binding protein 1* gene (*PABPC1*), was significantly downregulated by AR in an ADAR1-dependent manner. Of note, ectopic expression of circARSP91 could suppress HCC proliferation, tumor growth and invasion, highlighting the role of AR/ADAR1/circARSP91 axis in controlling HCC progression. However, the mechanism of how circARSP91 regulates HCC development needs to be further deciphered. Lin et al. [88] investigated the expression and function of a novel circRNA, circCDK13, in HCC development. CircCDK13 expression was decreased in HCC tumor tissues compared with that in corresponding adjacent normal liver tissues. The overexpression of circCDK13 significantly suppressed migratory and invasive abilities of HCC cells and inhibited cell cycle progression. CircCDK13 could modulate the genes that participate in Janus tyrosine kinase (JAK)/signal transducer and activator of transcription (STAT) and phosphoinositide 3-kinase (PI3K)/AKT signaling pathways. Moreover, circCDK13 overexpression markedly suppressed HCC progression in nude mice. Collectively, this study indicated that circCDK13 inhibited HCC development by regulating the JAK/STAT and PI3K/ATK signaling pathways.

The zinc finger family gene, *ZKSCAN1*, could be transcribed into both linear and circular (circZKSCAN1) forms of RNA in HCC [34]. The expression of both *ZKSCAN1* mRNA and circZKSCAN1 was markedly lower in HCC tissues compared with matched adjacent non-tumorous tissues. Inhibition of *ZKSCAN1* mRNA or circZKSCAN1 promoted cell proliferation, migration and invasion of HCC cells. In contrast, overexpression of *ZKSCAN1* mRNA or circZKSCAN1 had a suppressive effect on the migration and invasion of HCC cells. Inhibition or overexpression of both forms of RNAs didn’t interfere with each other. Surprisingly, high-throughput RNA sequencing approach together with bioinformatic analysis revealed a different molecular basis for the observed effects. *ZKSCAN1* mRNA played a regulatory role in cellular metabolism, such as retinol metabolism and phenylalanine metabolism. In contrast, circZKSCAN1 mainly mediated cancer-related pathways in HCC including the PI3K pathway, migration pathway and adhesion pathway. These results suggested a non-redundant role for *ZKSCAN1* circRNA and mRNA. Thus, *ZKSCAN1* mRNA and circZKSCAN1 tightly cooperated with each other to exert an anti-tumor effect on HCC. CircZKSCAN1 may serve as a diagnostic biomarker of HCC. 

**Figure 2:**
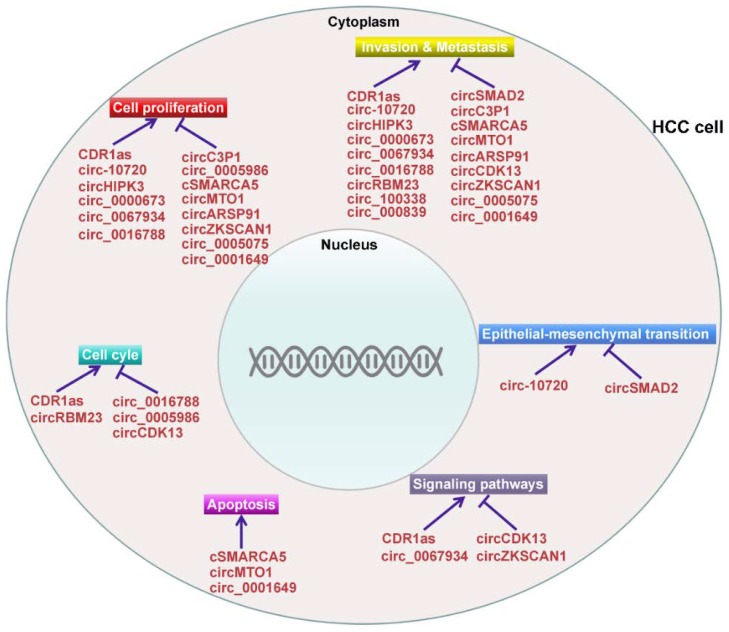
Function and mechanism of circRNAs in HCC carcinogenesis. Aberrantly expressed circRNAs in HCC can regulate cell proliferation, invasion and metastasis, epithelial-mesenchymal transition (EMT), cancer-related signaling pathways, cell apoptosis and cell cycle.

A number of studies have demonstrated the vital role of miRNAs in the occurrence and development of HCC [109,110,111]. CircRNAs can function as miRNA sponges to neutralize miRNA-mediated gene silencing [112]. Therefore, it is reasonable to assume that circRNAs are novel players involved in the carcinogenesis and development of HCC. Indeed, recent studies confirmed that circRNAs possessed regulatory function in HCC tumorigenesis [113,114,115] (Figure 2). Additionally, the circRNA/miRNA/mRNA pathway that regulates HCC pathogenesis has been uncovered, providing novel insights into the complex modulatory networks in the process of hepatocarcinogenesis. In-depth investigations on the function and mechanism of the circRNA/miRNA/mRNA pathway in HCC are required and will provide a new direction for diagnosing and treating HCC. Moreover, circRNAs could be used as molecular sponges to sequester oncogenic miRNAs/proteins, thereby abolishing their tumor-promoting abilities. Therefore, circRNAs may be promising novel therapeutic targets for HCC treatment.

### 4.3. CircRNAs Interfere with Hepatitis Virus Infection

Chronic hepatitis B virus (HBV) and hepatitis C virus (HCV) infections are most important risk factors for HCC [116]. Chronic HBV infection is the main cause of HCC in high incidence areas and accounts for at least 50% cases of primary liver tumors worldwide [117]. About 25% of HCC cases are attributable to HCV infection [118]. CircRNAs may regulate HBV or HCV infection, indirectly participating in hepatocarcinogenesis. Previously, a total of 99 dysregulated circRNAs were identified to be associated with chronic hepatitis B (CHB) [119]. CircRNA/miRNA interaction networks were constructed to predict the function of these circRNAs in CHB. Intriguingly, five circRNA/miRNA regulatory axes might be involved in pathways related to HBV infection, including “T cell receptor signaling pathway,” “Hepatitis B,” “Inflammatory mediator regulation of transient receptor potential (TRP) channels,” “TGF-β signaling pathway,” and “MAPK signaling pathway.” miR-122 is one of the most abundant miRNAs in the liver [120]. The liver specific miR-122 plays an important role in liver development, differentiation, homestasis, and metabolic functions [121]. miR-122 has been validated to target various genes involved in hepatocarcinogenesis, EMT, and angiogenesis, including cyclin G1, disintegrin and metalloproteinase domain–containing protein 10 (ADAM10), insulin-like growth factor-1 receptor (IGF1R), serum response factor (SRF), and Wnt1 [122]. In vivo studies demonstrated that inhibition of miR-122 could promote hepatocarcinogenesis, whereas restoration of miR-122 prevented HCC development [123,124,125]. Therefore, miR-122 serves as a tumor suppressor in the liver, and miR-122 mimics may represent a novel therapeutic strategy for HCC treatment. miR-122 is also required for the life cycle of HCV. miR-122 stabilized HCV RNA by directly binding to the 5′ UTR of the viral genome, thereby facilitating HCV replication [126]. Given its central role in the HCV life cycle, miR-122 holds great promise as a novel target for antiviral therapy. The efficacy of two anti-miR-122 drugs, Miravirsen and RG-101, was clinically tested [127,128]. Clinical proof-of-concept studies indicated that these miR-122 inhibitors efficiently decreased viral load in chronically infected HCV patients. Artificial circRNA sponges that efficiently absorbed miR-122 were previously produced [129]. Consequently, these artificial circRNAs could inhibit viral protein production, thereby blocking HCV replication. However, the concomitant suppression of the tumor-suppressive activity of miR-122 by artificial circRNAs should also be considered. CircRNA sponges may inhibit HCV replication but induce hepatocarcinogenesis by repressing miR-122 activity. Due to its essential roles in maintaining hepatic phenotype, loss of miR-122 may cause destructive consequences in patients. Before the miR-122-suppressive therapy could be used to combat HCV infection in clinical practice, the effects of miR-122 inhibition in HCC progression must be fully explored. Moreover, HCV-infected patients undergoing anti-miR-122 therapy must be routinely monitored for liver functions and cancer progression. More circRNA sponges can be devised to target miRNAs that play an important role in hepatitis virus replication and pathogenesis. Given their stable structures and cellular localization, circRNAs may serve as an interesting tool for applications in molecular medicine.

Emerging evidence indicated that chronic HBV/HCV infection changed miRNA expression profiles, and the dysregulated miRNAs performed significant functions in viral replication and the occurrence of virus-related HCC [130,131,132]. CircRNAs may function as important regulators in tumorigenesis and progression of HCC by sponging miRNAs involved in HBV/HCV infection–induced hepatocarcinogenesis. In addition, recent studies have indicated that circRNAs play a crucial role in controlling antiviral immune responses [133,134]. CircRNAs may participate in the pathogenesis of hepatitis virus–associated HCC by exerting their regulatory effects on host immune system. Nevertheless, there is little knowledge about the relationship between circRNAs and HBV/HCV infection, limiting the comprehensive elucidation of the molecular mechanisms underlying hepatitis virus infection–induced hepatocarcinogenesis. More detailed research is required to uncover the functional role of circRNAs in the pathogenesis of hepatitis virus–associated HCC.

### 4.4. CircRNAs May Serve as Potential Diagnostic Biomarkers for HCC

As circRNAs are evolutionally conserved, abundant, and stable in the cytoplasm, they may hold great potentials for cancer diagnosis [27,32]. The potential values of circRNAs in HCC diagnosis have been disclosed. A total of 61 differentially expressed circRNAs were identified in HCC tissues compared with adjacent normal tissues through circRNA microarray analysis [90]. Among these differentially expressed circRNAs, hsa_circ_0005075 displayed markedly difference in expression between HCC and normal tissues. Hsa_circ_0005075 exhibited significant association with the clinicopathological parameters of HCC patients and possessed a great diagnostic potential with a high degree of accuracy, sensitivity, and specificity. Experimental verification confirmed that hsa_circ_0005075 was highly expressed in HCC tissues and could inhibit the activity of miR-431 [91]. Moreover, inhibition of hsa_circ_0005075 promoted cell apoptosis but suppressed cell proliferation, migration and invasion of HCC cells. This result highlighted the fact that hsa_circ_0005075 represented a promising biomarker for HCC diagnosis. 

Hsa_circ_0001649 expression was shown to be significantly lower in HCC tissues compared with paired adjacent non-tumorous tissues, and its expression levels associated with tumor size and the occurrence of tumor embolus in HCC [92]. Hsa_circ_0001649 might be involved in tumorigenesis and metastasis of HCC by sponging several miRNAs including miR-1283, miR-182-3p, miR-1972, miR-4310, miR-4502, miR-6511b-5p, miR-6811 and miR-888-3p. ROC analysis indicated that hsa_circ_0001649 expression could be utilized as a biomarker for differentiating HCC tissues from adjacent non-tumorous liver tissues. Additionally, HCC patients with low hsa_circ_0001649 expression had significantly reduced survival time than those with high hsa_circ_0001649 expression. Functional analysis demonstrated that hsa_circ_0001649 overexpression inhibited HCC cell proliferation and promoted cell apoptosis, and also restrained the migratory and invasive capabilities of HCC cells [93]. Conversely, knockdown of hsa_circ_0001649 in HCC cells could increase the expression of matrix metallopeptidases (MMPs) that play a promotive role in HCC metastasis. These findings indicated that hsa_circ_0001649 was a potential predictive biomarker for HCC, with relatively high degrees of specificity, accuracy, and sensitivity.

Recently, a global circRNA expression was unraveled using a circRNA microarray in HCC patients [135]. Hsa_circ_0128298 was shown to be significantly upregulated in HCC tissues. High expression of hsa_circ_0128298 was correlated with clinicopathological parameters including intrahepatic metastasis, lymphatic invasion, and organ metastasis in HCC patients. ROC and Cox regression analyses suggested that hsa_circ_0128298 had potential diagnostic and prognostic values for HCC. Moreover, Kaplan-Meier analysis demonstrated that HCC patients with lower hsa_circ_0128298 expression levels had prolonged overall survival compared to those with higher hsa_circ_0128298 expression levels. In summary, hsa_circ_0128298 might participate in the proliferation and metastasis of HCC and represented a potential diagnostic biomarker and prognostic indicator in HCC. Fu et al. [136] also revealed the circRNA expression profiles in HCC tissues and paired para-tumorous tissues and further explored their clinical significances and relevant mechanisms for HCC progression. As a result, a total of 527 differentially expressed circRNAs were identified in HCC tissues, of which 174 were upregulated and 353 were downregulated. Hsa_circ_0004018, one of the most downregulated circRNAs, was correlated with clinicopathological factors in HCC patients, such as serum α-fetoprotein (AFP) levels, tumor-node-metastasis (TNM) stage, and tumor size and differentiation degree. Hsa_circ_0004018 was predicted to be implicated in cancer-related pathways including “Transcriptional misregulation in cancer,” “Central carbon metabolism in cancer,” “Pathways in cancer,” and “Viral carcinogenesis” by acting as miRNA sponges. The evaluated diagnostic performance along with its tumor tissue-specific expression pattern underlined that hsa_circ_0004018 could be used as a diagnostic marker to distinguish HCC tissues from non-cancerous tissues.

Yao et al. [137] found that hsa_circ_0068669 expression was significantly decreased in HCC tissues compared with matched non-tumorous tissues. Hsa_circ_0068669 expression was correlated with MVI and TNM stage. These findings demonstrated the potential value of hsa_circ_0068669 as a biomarker for HCC metastasis. Hsa_circ_0003570 was the first found downregulated circRNA in HCC [138]. Decreased expression of hsa_circ_0003570 was significantly correlated with the clinicopathological characteristics of HCC patients including tumor size, differentiation, MVI, TNM stages, and serum AFP levels. These results demonstrated that hsa_circ_0003570 might be involved in HCC invasion and metastasis and could be used as a diagnostic biomarker for HCC tumorigenesis. The diagnostic values of circZKSCAN1 have also been studied [34]. The expression levels of circZKSCAN1 were found to be correlated with tumor numbers, cirrhosis, MVI, vascular invasion and tumor degree. ROC analysis indicated that circZKSCAN1 might be a useful biomarker for effective discrimination of HCC tissues from non-tumorous tissues.

It is believed that HCC can be cured if diagnosed at an early stage. The early diagnosis of HCC in patients is of paramount importance. The field of early HCC diagnosis has long been a research focus of scientists. As currently used biomarkers and tools usually fail to diagnose HCC at an early stage, early diagnosis of HCC remains a major clinical challenge. Therefore, it is urgent to identify efficient biomarkers for HCC diagnosis. Over the past decades, an increasing awareness of circRNAs has made researchers pay attention to the potential role of circRNAs in clinical diagnosis of HCC. CircRNAs are insensitive to ribonucleases due to their stable circular structures. The expression of circRNAs is shown to be associated with the traditional biomarkers including AFP. Previously, the diagnostic potential of hsa_circ_0001445 for HCC detection was evaluated [85]. The plasma levels of hsa_circ_0001445 were detected in HCC patients, cirrhosis patients, hepatitis B patients, and healthy controls. The results showed that the transcriptional levels of plasma hsa_circ_0001445 in HCC patients were dramatically lower than those in healthy controls, cirrhosis patients, and hepatitis B patients. The plasma hsa_circ_0001445 levels in cirrhosis and hepatitis B patients were lower than those in healthy controls. However, no significant difference in the transcriptional levels of plasma hsa_circ_0001445 was found between cirrhosis patients and hepatitis B patients. Thus, the plasma hsa_circ_0001445 might be used to differentiate HCC patients from healthy controls, cirrhosis or hepatitis B patients. Correlation analysis demonstrated that plasma hsa_circ_0001445 levels were strikingly lower in HCC patients with low-level serum AFP than those in HCC patients with high-level serum AFP. Further analysis indicated that plasma hsa_circ_0001445 levels were correlated with serum AFP levels in HCC patients, whereas no statistically significant association was detected between hsa_circ_0001445 and other biochemical indices such as alanine aminotransferase (ALT) and aspartate aminotransferase (AST). Although AFP serves as one of the most commonly used biomarkers for HCC diagnosis, the low specificity and sensitivity of this biomarker have limited its application in clinical settings. In addition, the combined diagnostic values of plasma hsa_circ_0001445 and serum AFP were also analyzed. Surprisingly, the efficacy of the combined diagnosis in distinguishing HCC patients from healthy controls as well as patients with cirrhosis or hepatitis B was higher than that using serum AFP levels or plasma hsa_circ_0001445 levels alone. These results indicated that the plasma hsa_circ_0001445 might serve as a novel diagnostic biomarker for HCC.

CircRNAs also exhibit a high degree of HCC tissue-specificity. Moreover, some circRNAs may be correlated with tumor size, TNM stage, and metastasis in HCC patients, reflecting the stage characteristics of HCC tumorigenesis. Therefore, circRNAs may be efficient novel biomarkers for HCC diagnosis and hold great potential to be used in the clinical diagnosis of HCC. Nevertheless, before circRNAs could be used as effective biomarkers for early HCC diagnosis, several important issues must be addressed. Although several circRNAs display good diagnostic performances in differentiating HCC tissues from non-cancerous tissues, the efficiency and reliability of using circRNAs for HCC diagnosis in clinical practice need to be proven. Currently, the detection of circRNAs in HCC mainly focuses on tissue samples of patients. More easily acquired and non-invasive clinical samples (e.g., blood, saliva, urine, etc.) should be detected for circRNA expression in future studies. CircRNAs can be detected in body fluids, such as human cell–free plasma and saliva [139,140,141,142]. Many circRNAs are abundantly expressed in the blood while their linear counterparts exhibit low abundances, paving ways for further identification of novel cancer biomarkers in body fluids. Specifically, exosomes, critical components of cellular communication, have been found in diverse body fluids including blood [143], urine [144], saliva [145], and cerebrospinal fluid [146]. CircRNAs can be transferred from cells to exosomes [147]. CircRNAs are found to be located in cell-derived exosomes as well as tumor-derived exosomes. Moreover, circRNAs are enriched and highly stable in exosomes due to their characteristics [148]. The exosomes that contain circRNAs may be largely released by tumor cells, protecting circRNAs from ribonuclease-mediated degradation in extracellular fluids. More importantly, tumor-derived exosomal circRNAs could enter the circulation and be readily measured in the serum [149]. Exosomal circRNAs were able to distinguish patients with colon cancer from healthy controls [150], demonstrating their importance as potential non-invasive biomarkers for diagnostic purposes. Therefore, exosomal circRNAs may represent promising biomarkers that would reduce the use of invasive surgery for cancer detection [151]. The exosomal circRNAs correlated with HCC require to be further elucidated. Further studies on exosomal circRNA would lay the foundation for development of circRNAs as a novel class of exosome-based biomarkers for HCC diagnosis. The sample size/quantity and processing method, detection approach uniformity, and cut-off value ascertainment need to be optimized for developing circRNAs as perfect biomarkers for HCC diagnosis. Combined detection of circRNAs and traditional biomarkers may also be considered to improve the diagnostic efficiency in HCC.

## 5. Conclusions

HCC is one of the most predominant subjects of liver malignancies, which causes a major health problem for a long time. Although new advanced therapeutic approaches were successively carried out in the last few years, the survival rate of HCC is still low. Accumulating evidence indicates that altered circRNA expression can affect the tumorigenesis and progression of HCC, and these circRNAs exhibit great potentials in HCC diagnosis, therapy, and prognosis. Research aimed at revealing the mechanisms underlying the effect of circRNAs on HCC carcinogenesis has indicated that circRNAs function as miRNA sponges to regulate the expression of genes/proteins involved in cell cycle, proliferation, invasion, and metastasis. Based on previous studies, circRNAs are undoubtedly implicated in the onset and development of HCC. CircRNAs possess multiple advantages including high abundance and stability, suggesting that circRNAs are ideal diagnostic biomarkers and promising therapeutic targets for HCC. However, compared with other ncRNAs such as miRNAs and lncRNAs, the study of circRNAs in HCC is still in its infancy. So far, only a small quantity of functional circRNAs have been discovered and characterized in HCC. These circRNAs function to regulate HCC progression generally through their miRNA sponge function. It is possible that circRNAs participate in HCC tumorigenesis by different mechanisms, such as competing with linear splicing of pre-mRNAs that are transcribed from tumor suppressor genes or encoding proteins that function as tumor promoters. Therefore, the features of circRNAs including their biogenesis, degradation, locations, and functions remain to be elucidated. In addition, more detailed studies are required to comprehensively disclose the molecular mechanisms underlying the functional role of circRNAs in HCC pathogenesis. In-depth investigations on the function and mechanism of circRNAs in HCC would enrich our knowledge of the complex regulatory networks involved in hepatocarcinogenesis. Moreover, further understanding of the relationship between circRNAs and HCC aetiology will accelerate the clinical application of circRNAs in HCC diagnosis and therapy.

## Figures and Tables

**Table 1:** Overview of deregulated circRNAs in hepatocellular carcinoma (HCC).

CircRNA	Alias	Gene Symbol	Chromosome	Expression Change	Function	Possible Mechanism	Reference
CDR1as (ciRS-7)	hsa_circ_0001946	CDR1	chrX	Up	proliferation (+); invasion (+)	miRNA sponge	[73,74,75]
Cul2 circRNA	circ-10720	CUL2	chr10	Up	proliferation (+); migration (+); invasion (+); EMT (+)	miRNA sponge	[76]
circHIPK3	hsa_circ_0000284	HIPK3	chr11	Up	proliferation (+); migration (+)	miRNA sponge	[36]
hsa_circ_0000673	-	RSL1D1	chr16	Up	proliferation (+); invasion (+)	miRNA sponge	[77]
hsa_circ_0067934	-	PRKC1	chr3	Up	proliferation (+); migration (+); invasion (+)	miRNA sponge	[78]
hsa_circ_0016788	-	TRIM11	chr1	Up	proliferation (+); invasion (+); apoptosis (−)	miRNA sponge	[79]
circRBM23	-	RBM23	chr14	Up	viability (+); migration (+)	miRNA sponge	[80]
hsa_circ_100338	-	SNX27	chr1	Up	migration (+); invasion (+)	miRNA sponge	[81]
circSMAD2	hsa_circ_0000847	SMAD2	chr18	down	migration (−); invasion (−); EMT (−)	miRNA sponge	[82]
circC3P1	-	C3P1	chr19	down	proliferation (−); migration (−); invasion (−)	miRNA sponge	[83]
hsa_circ_0005986	-	PRDM2	chr1	down	proliferation (−)	miRNA sponge	[35]
cSMARCA5	hsa_circ_0001445	SMARCA5	chr4	down	proliferation (−); migration (−); invasion (−); apoptosis (+)	miRNA sponge	[84,85]
circMTO1	hsa_circ_0007874/hsa_circ_104135	MTO1	chr6	down	proliferation (−); invasion (−); apoptosis (+)	miRNA sponge	[86]
circARSP91	hsa_circ_0085154	PABPC1	chr8	down	proliferation (−); invasion (−)	regulated by ADAR1	[87]
circCDK13	hsa_circ_0001699	CDK13	chr7	down	migration (−); invasion (−)	regulating JAK/STAT and PI3K/ATK signaling pathways	[88]
circZKSCAN1	hsa_circ_0001727	ZKSCAN1	chr7	down	proliferation (−); migration (−); invasion (−)	regulating cancer-related pathways	[34]
hsa_circ_000839	hsa_circ_0000497	SLAIN1	chr13	up	migration (+); invasion (+)	regulated by miR-200b	[89]
hsa_circ_0005075	-	EIF4G3	chr1	up	proliferation (−); migration (−); invasion (−)	miRNA sponge	[90,91]
hsa_circ_0001649	-	SHPRH	chr6	down	proliferation (−); migration (−); invasion (−); apoptosis (+)	Promoting MMPs expression	[92,93]
